# Complex Recombination Landscape and Lineage Turnover in Classical Human Astroviruses

**DOI:** 10.3390/microorganisms14040857

**Published:** 2026-04-10

**Authors:** Yulia Aleshina, Vladimir Frantsuzov, Alexander Lukashev

**Affiliations:** 1 Martsinovsky Institute of Medical Parasitology, Tropical and Vector Borne Diseases, Sechenov First Moscow State Medical University, 119435 Moscow, Russia; alexander_lukashev@hotmail.com; 2 Faculty of Bioengineering and Bioinformatics, Lomonosov Moscow State University, 119234 Moscow, Russia; 3 Department of Normal Physiology, Sechenov First Moscow State Medical University, 119435 Moscow, Russia; yakrit2013@yandex.ru

**Keywords:** human astroviruses, enteric viruses, recombination, recombinant forms, evolution

## Abstract

Human astroviruses are small, non-enveloped RNA viruses belonging to the family *Astroviridae*. Among the four species known to infect humans, the species *Mamastrovirus hominis* (the classical human astroviruses, formerly MAstV1) is associated with gastrointestinal illness worldwide, while three more recently identified species have been linked to lethal central nervous system infections. High substitution rates and recombination drive their rapid evolution, yet recombination patterns in classical human astroviruses remain poorly characterized. This study systematically analyzes patterns and temporal dynamics of natural recombination in classical human astroviruses. Publicly available genomes of classical human astroviruses were analyzed to identify recombination hotspots. Recombinant forms were defined as stable phylogenetic lineages unaffected by recombination, and their half-lives were estimated based on time-scaled phylogenies (BEAST2v2.7.7). Recombination in classical human astroviruses occurred most frequently at the ORF1b/ORF2 junction, but also within ORF1a, at the ORF1a/ORF1b junction, and within ORF2. Only the 3′-part of ORF1a and a fragment of ORF1b exhibited robust temporal signal, yielding substitution rates of 2.35 × 10^−3^ and 2.14 × 10^−3^ s/s/y, respectively. The half-lives of recombinant forms varied considerably by genomic region: longest for exchanges between the parts of ORF1a (21 years), intermediate for ORF1a/ORF1b recombinants (7–9 years), and shortest for ORF1ab/ORF2 recombinants (2.5–3.6 years). The estimated half-lives for recombinants align with those reported for human enteroviruses and noroviruses. These findings highlight the dynamics of the generation of astrovirus diversity and may inform advanced surveillance of emerging strains.

## 1. Introduction

Human astroviruses are small non-enveloped RNA viruses that belong to the family *Astroviridae*. Four species of human astroviruses are recognized to date. Viruses belonging to the species *Mamastrovirus hominis* (MAstV1), also referred to as classic astroviruses, were discovered in 1975 and are associated with gastrointestinal illness worldwide [1,2]. Based on antigenic properties, classic human astroviruses are classified into eight serotypes, HAstV1-HAstV8 [1]. The rest of the human-infecting species—*Mamastrovirus melbournense* (MAstV6), *Mamastrovirus homustovis* (MAstV8), *Mamastrovirus virginiaense* (MAstV9)—were identified in the late 2000s, along with human astroviruses VA3-VA6 and MLB2-MLB3, which remain unclassified to species level. While their pathogenicity has not yet been explicitly established, these species are known to be associated with lethal central neural system infections [3].

Astroviruses have a single stranded positive-sense RNA genome ranging from 6.7 to 7.0 kb in length. The genome is covalently linked at the 5′ end to viral genome-linked protein (VPg), which is essential for virus infectivity [4], and polyadenylated at the 3′ untranslated region. Astrovirus genome contains three main open reading frames (ORFs): ORF1a encodes a nonstructural polyprotein (nsP1a) that includes VPg and serine protease, while ORF1b and ORF2 encode RNA-dependent RNA polymerase (RdRp) and capsid proteins, respectively. ORF1b is expressed through programmed ribosomal frameshifting, which occurs at a conserved AAAAAAC sequence within the ORF1a/ORF1b overlap region [5]. ORF2 partially overlaps with ORF1b and is translated from a subgenomic RNA. Most human astroviruses have an additional ORF named ORFX that overlaps with ORF2 in the +1 reading frame and encodes viroporin. Notable exceptions include *M. virginiaense* (VA1), *M. homustovis* (VA2), and unclassified VA isolates (VA3–VA6) [6].

Similar to other positive-sense RNA viruses, classical human astroviruses evolve rapidly due to mutations and recombination. The published evolutionary rate estimates vary from 4.5 × 10^−4^ substitutions/site/year in the capsid-encoding gene [7] to 3.7 × 10^−3^ in the other genome fragments [8]. Homologous recombination occurs when divergent viruses co-infect a single cell, resulting in large-scale genomic changes. In mammalian astroviruses, recombination is frequent [9,10,11,12,13,14,15,16,17,18,19,20,21,22], but occurs strictly within a virus species. Therefore, the capacity for recombination has been proposed as an additional criterion for species demarcation, alongside with pairwise genetic distances in the ORF1b and ORF2 regions and host species [23]. Recombination plays a key role in the generation of novel astrovirus strains, which are reported regularly and may arise from exchanges between viruses of the same or different genotypes/serotypes (Table 1). Although recombination occurs most commonly at the ORF1b/ORF2 junction, events at the ORF1a/ORF1b junction and within the open reading frames have also been documented, including triple recombinants [15,24,25]. It is noteworthy that recombination within ORF2 was reported close to the ends of regions encoding individual proteins and spared their cores.

Recombination has been reported between human astroviruses and astroviruses from other hosts—including feline [20,26], porcine [27], and California sea lion [28]. However, it may be hard to distinguish recombination events that occurred in ancestral viruses that later became established in distinct host species from a true recombination between viruses infecting distinct species. In any case, there is no evidence that such recombination is routine and ubiquitous as observed among classical human astroviruses.

This study aims to systematically investigate natural recombination in classical human astroviruses and determine the frequency at which novel recombinant variants emerge and become established in circulation.

**Table 1:** Recombinant classical human astroviruses reported previously. Astrovirus serotypes and genotypes are indicated as in the original publication.

Location of Recombination Breakpoint	Recombinant Virus	Potential Parents (the Genome Regions Derived from Parents Are Indicated in Brackets)	Reference
Within ORF1a	HAstV1	HAstV7 (5′-part of ORF1a), HAstV3 (3′-part of ORF1a)	[14]
	* HAstV4	HAstV3 (ORF1a), HAstV1 (ORF1b)	[15]
ORF1a/ORF1b junction	* HAstV1	HAstV5 (ORF1a), HAstV8 (ORF1b)	[24]
	* HAstV2c	HAstV6 (ORF1a), HAstV3 (ORF1b)	[15]
	* HAstV2b	HAstV5 (ORF1a), HAstV4 (ORF1b)	
ORF1b/ORF2 junction	HAstV1	HAstV8 (ORF1b), HAstV1 (ORF2)	[12,29]
	HAstV1	HAstV8 (ORF1a), HAstV1 (ORF2)	[30]
	* HAstV1	HAstV8 (ORF1b), HAstV1 (ORF2)	[24]
	HAstV2a	Ambiguous (ORF1ab), HAstV2 (ORF2)	[15]
	HAstV2d	HAstV1b (ORF1b), HAstV2d (ORF2)	
	HAstV2	HAstV3 (ORF1b), HAstV2 (ORF2)	[14,15,29]
	HAstV2	HAstV6 (ORF1b), HAstV2 (ORF2)	[12]
	* HAstV2b	HAstV5 (ORF1ab), HAstV4 (ORF2)	[15]
	* HAstV2c	HAstV3a (ORF1b), HAstV2c (ORF2)	[9]
	HAstV2	HAstV8 (ORF1a), HAstV2 (ORF2)	[30]
	HAstV3c	HAstV1 (ORF1b), HAstV3 (ORF2)	[10]
	HAstV3	HAstV2 (ORF1a), HAstV3 (ORF2)	[30]
	HAstV3	HAstV8 (ORF1b), HAstV3 (ORF2)	[29]
	* HAstV4	HAstV1 (ORF1b), HAstV4 (ORF2)	[15]
	HAstV5	HAstV3 (ORF1b), HAstV5 (ORF2)	[13]
	HAstV8	HAstV2 (ORF1b), HAstV8 (ORF2)	[12]
	HAstV8	HAstV2 (ORF1ab), HAstV8 (ORF2)	[11]
Within ORF1b	HAstV4	HAstV1 (ORF1a, 5′-half of ORF1b), HAstV4 (3′-half of ORF1b, ORF2)	[15]
	* HAstV8	HAstV8, HAstV1, HAstV2	[25]
Within ORF2	HAstV2c-2b	HAstV2c (5′-part), HAstV2b (3′-part)	[9]
	HAstV1a-1d	HAstV1a (5′part), HAstV1d (3′-part)	[31]
	HAstV1b	Heterologous non-HAstV RNA (5′-end of VP34), HAstV1b (the rest of ORF2)	
	HAstV3a	HAstV3 (5′-part), HAstV3a (3′-part)	
	HAstV5a	HAstV5a/GO-12 (5′-part), HAstV5a/Oxford-S5 (3′-part)	

* triple recombinants.

## 2. Materials and Methods

### 2.1. Data Collection

Complete genome sequences of the species *Mamastrovirus hominis* (formerly *Mamastrovirus 1*) (*N* = 125) were collected from GenBank database as of March 2024 using the query: “Mamastrovirus 1”[porgn] AND (“6000”[SLEN]: “8000”[SLEN])”. Viral metadata, including isolate, strain, isolation source, country of isolation, and collection date, were extracted from the corresponding qualifiers in GenBank entries using a custom Python script and manually curated. Missing sampling dates could be retrieved from associated publications for 7 out of 17 incomplete records.

Coding sequences (CDS)—specifically ORF1a, ORF1b, and ORF2—were extracted from complete genomes according to the coordinates indicated in GenBank records and manually verified. The CDS were translated to amino acid sequences, which were then aligned separately using the ClustalW algorithm in MEGA v11 [32]. The resulting alignments were back-translated to produce codon-based alignments. Records lacking CDS coordinates were discarded. Where possible, missing coordinates of ORFs were inferred using similar sequences from the same study; this curation resulted in the exclusion of only 7 sequences. Sequences isolated from sewage exhibited many insertions showing no homology to other sequences in the datasets and were excluded from the analysis. The resulting alignment of complete genomes comprised 104 sequences.

Genomes containing > 5 ambiguous nucleotides (or >3 consecutive ambiguities), or >50 gaps in a row, were discarded, and ambiguous sites were in a few cases resolved by selecting the most probable base from the best-matching BLAST v2.16.0 hit [33] within a 100-nt window (E-value threshold ≤ 1 × 10^−10^), using a custom script (https://github.com/v-julia/resolve_ambiguous; accessed on 9 April 2026). After quality control, the dataset comprised 77 sequences (Table S1). For recombination analyses, alignments of ORF1a, ORF1b, and ORF2 were concatenated.

To verify the serotype assignment of individual sequences, a preliminary Maximum Likelihood (ML) phylogenetic tree was constructed for the capsid-encoding region (ORF2) using the IQ-TREE web server [34]. For sequences with serotype not indicated in Genbank entry, the serotype was inferred based on phylogenetic grouping.

### 2.2. Recombination Analysis

A full exploratory recombination scan was performed to identify recombination breakpoints using RDP4 v101 software [35] applying nine standard recombination detection methods: RDP [36], GENECONV [37], MaxChi [38], Chimaera [39], BootScan [40], SiScan [41], 3seq [42], PhylPro [43], and LARD [42]. Only events supported by at least 5 methods were considered. Putative events were further validated by manually examining the phylogenetic placement of recombinant sequences and their parents.

The overall recombination patterns were visualized using breakpoint distribution plot (sliding window 200 nt, step size 100 nt) and recombination region count matrices. Recombination region count matrices indicate the number of times the detected recombination events separated that specific pair of genome positions.

Breakpoint distribution was inspected to identify regions with minimal evidence of recombination. Subsequently, these recombination-free regions were excised from the alignments for downstream phylogenetic analysis.

### 2.3. Phylogenetic Analysis

ML phylogenetic trees for recombination-free regions were built on the IQ-TREE web server (auto-detection of substitution model, ultrafast bootstrap analysis with 1000 replicates) [34,44,45].

The preliminary assessment of temporal signal for each recombination-free region was performed using TempEst [46]. The best-fitting tree root was selected using “heuristic residual mean squared” function. Root-to-tip divergence and sampling dates were exported from TempEst; linear regression was then performed in Python v3.10 (scikit-learn, SciPy v1.15.3 packages). A recombination-free region was considered to have a temporal signal if (i) the correlation between root-to-tip divergence and sampling date was positive and (ii) the *p*-value of the regression slope was <0.05. The correspondence between root-to-tip divergence and sampling dates was visualized with the Matplotlib v3.10.3 Python package, using the exported TempEst data.

For genome regions that exhibited a temporal signal in TempEst, time-calibrated phylogenies were inferred using BEAST v2.7.7 [47]. First, the best-fitting nucleotide substitution models and partitioning schemes were selected using PartitionFinder 2.1.1 software [48] under the AIC criterion and with substitution models restricted to those implemented in BEAST v.2.7.7. Marginal likelihoods were then estimated for every combination of molecular clock models (strict, relaxed lognormal, relaxed exponential) and tree prior models (coalescent constant population, exponential population, and bayesian skyline) using Nested Sampling plugin in BEAST2 [49]. Along with marginal likelihood, Nested sampling provides estimates of standard deviation of marginal likelihood. If the difference between marginal likelihoods of two models was less than 2×sqrt(SD1×SD1 + SD2×SD2), the analysis was repeated with more particles to ensure that the difference was not due to randomization. The combination with the highest marginal likelihood was selected as the best-fitting model; Bayes factors (BF) were calculated for pairwise comparisons (Table S2).

The final assessment of the temporal signal was performed using Bayesian Estimation of Temporal Signal (BETS) [50,51]. Under the best-fitting model identified above, marginal likelihoods were estimated for two models—one incorporating the actual sampling times and another suggesting all samples to be contemporaneous (sampling dates set to zero). If log Bayes factor was >5 in favor of the model with actual sampling dates, the dataset of genome regions was interpreted as having a robust temporal signal. Only regions satisfying this criterion were used for full MCMC analysis.

For alignments of genome regions with temporal signal confirmed by BETS, five independent Markov chain Monte Carlo (MCMC) runs were performed to assess the convergence. Chain lengths and sampling frequencies were adjusted per dataset (see Table S2). MCMC convergence was verified in Tracer v1.7.2 [52]; all parameters achieved effective sample size > 200. The resulting posterior tree distributions from independent runs were combined using LogCombiner. Maximum Clade Credibility (MCC) trees were then summarized from the combined files using TreeAnnotator v.2.7.7. after discarding 10% of each chain as burn-in.

The phylogenetic trees were visualized in R environment using ggtree package [53].

### 2.4. Temporal Dynamics of Recombination

To characterize the turnover of recombinant lineages, we estimated the half-life of recombinant forms—the median period during which a lineage arising from a distinct recombination event persists in the host population before it is no longer detectable. A recombinant form is defined here as a viral lineage descending from a single ancestral recombination event between two distinguishable parental genomes, with no subsequent detectable recombination.

We employed a phylogenetic tree comparison approach previously applied to enteroviruses, noroviruses [54,55], and to foot-and-mouth disease virus [56]. This method identifies recombinant forms by comparing time-calibrated phylogenies inferred from genomic regions that are frequently exchanged through recombination. The lifetime of each recombinant form is measured as the interval between the estimated time of its most recent common ancestor (tMRCA), marking its emergence, and the isolation date of its most recently sampled descendant. The recombinant half-life for a group of viruses is then taken as the median of lifetimes of individual recombinant forms.

In earlier studies [54,55], tree comparisons were performed manually. To overcome this limitation, we previously developed RF-HL, a Python tool that automates the identification of coinciding clades and the calculation of recombinant half-lives [56]. RF-HL requires two input trees: a reference time-scaled phylogeny (Nexus format, e.g., from BEAST) that is used for dating clades and a phylogeny inferred by any other method (Newick or nexus format). It implements two complementary detection strategies—common subtree search and common bipartition search—and outputs the matching subtrees together with the estimated lifetimes of all detected recombinant forms. A detailed description of the algorithm and its validation is provided in [56]. The tool is freely available at https://github.com/v-julia/RF_HL (Accessed on 9 April 2026).

Half-life time calculations were performed under two scenarios. When both genomic regions exhibited robust temporal signal (3′-part of ORF1a and ORF1b), two Bayesian time-scaled phylogenies were compared. When only one region possessed a temporal signal, its Bayesian phylogeny was compared to a maximum likelihood phylogeny of the partner region.

## 3. Results

### 3.1. Identification of Recombination Patterns in Classical Human Astroviruses

The refined dataset of complete genomes of classical human astroviruses comprised 77 sequences and included all serotypes, except for serotype 7 (Table 2). Serotype 1 was the most prevalent (*N* = 32), followed by serotype 4 (*N* = 19), while the other serotypes were represented by less than 10 sequences.

Recombinant human astroviruses have been detected in field studies (Table 1). Most recombinants exhibited breakpoints at the ORF1b/ORF2 junction, although recombinants with breakpoints within ORFs have also been reported. For many of the detected recombinants, complete genome sequences were not available. To estimate recombination prevalence across the genome and identify genome regions that were more frequently exchanged and contributed to the generation of recombinant forms, we conducted an exploratory analysis using the RDP4 program. A permutation test was performed to compare the observed incidence of breakpoints with a random distribution, corrected for sequence similarity.

**Figure 1:**
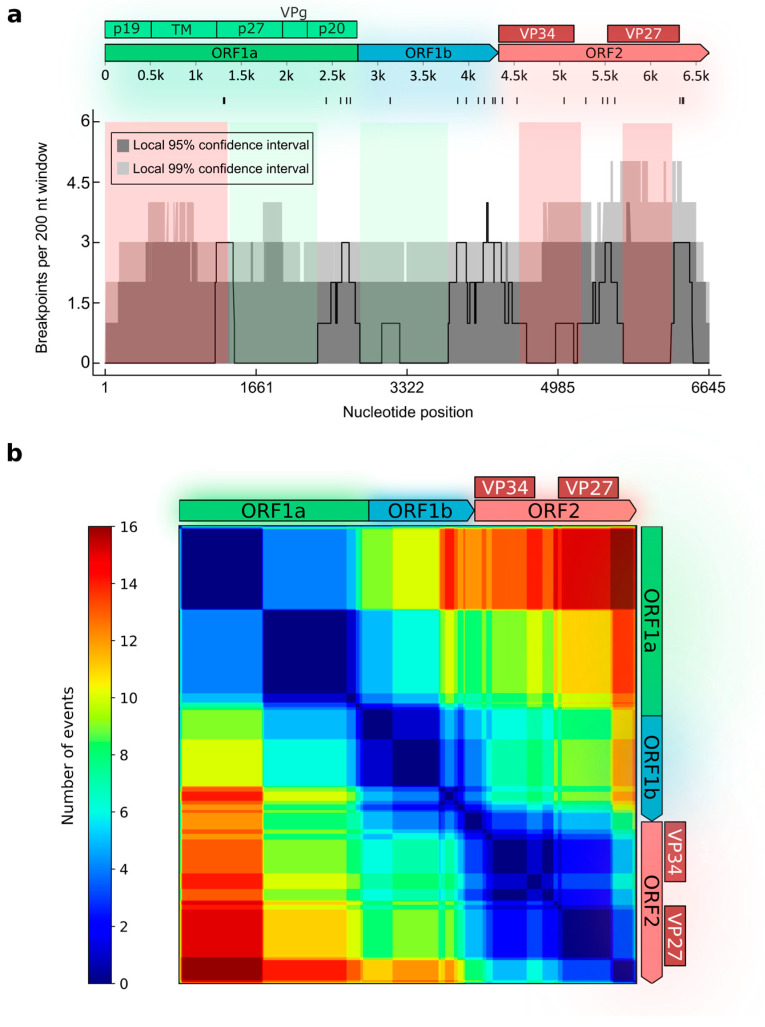
Recombination analysis of classical human astroviruses. The analysis was performed in RDP4 v101 software [35]. (**a**) Distribution of recombination breakpoints across astrovirus ORFs. A schematic of the astrovirus open reading frames (ORFs) and mature proteins are shown at the top (TM—transmembrane protein). Individual breakpoint positions are indicated by tick marks below this schematic. The lower panel displays the density of detected breakpoints (solid black line) calculated using a 200-nt sliding window with a 10-nt step. The light and dark gray areas represent the local 95% and 99% confidence intervals, respectively, generated from 1000 permutations. Regions where the observed recombination event count exceeds these confidence intervals represent significant recombination hotspots. Genome regions selected for subsequent temporal analysis are highlighted: those with a temporal signal are shaded green, while those without are shaded red. Nucleotide positions are shown in relation to OR371570 (HAstV4) sequence. (**b**) Recombination region count matrix. Each cell of the matrix represents a pair of nucleotide positions along the genome; the color scale (from blue to red) indicates the number of unique recombination events (mapped in panel (**a**)) partitioning that specific site pair. Warmer colors indicate genomic regions more frequently exchanged through recombination.

A total of 22 unique recombination events, supported by more than five methods and confirmed via manual inspection, were detected in the dataset (Figure 1). The permutation test identified a single recombination hotspot located within approximately 500 nucleotides around the ORF1b/ORF2 junction (8 events) (Figure 1a). Although breakpoint densities in other regions did not reach statistical significance as defined by the permutation test (i.e., they fell below the 99% local confidence interval threshold), the distribution of recombination breakpoints was not uniform. Clusters of breakpoints with densities approaching the upper bound of the confidence interval were observed in three regions: within ORF1a, at the beginning of the region encoding the serine protease p27 (positions 1301–1327 of the reference sequence OR371570/HAstV4); near the ORF1a/ORF1b junction (within the p20 protein); and within ORF2, where breakpoints clustered in the middle of the gene (approximately between the VP34 and VP25/VP27 encoding regions) and in the 3′ end of ORF2 (corresponding to the start of the acidic C-terminal domain).

Recombination region count matrices, which indicate the number of times unique recombination events separate genome regions, confirmed the division of ORF1ab into three blocks with no recombination within them: the 5′-half of ORF1a, the 3′-half of ORF1a, and ORF1b (Figure 1b). Within ORF2, two blocks with no evidence of internal recombination were identified, corresponding to the part of VP34 and the part of VP25/27. The matrices further revealed that recombination breakpoints within ORF1a and at the ORF1a/ORF1b junction contribute to the exchange of ORF1a relative to the ORF2 region. As a result, ORF1a was found to be recombinant relative to ORF2 more frequently than ORF1ab as a whole (Figure 1b).

### 3.2. Evaluating Temporal Signal Across the Astrovirus Genome

**Figure 2:**
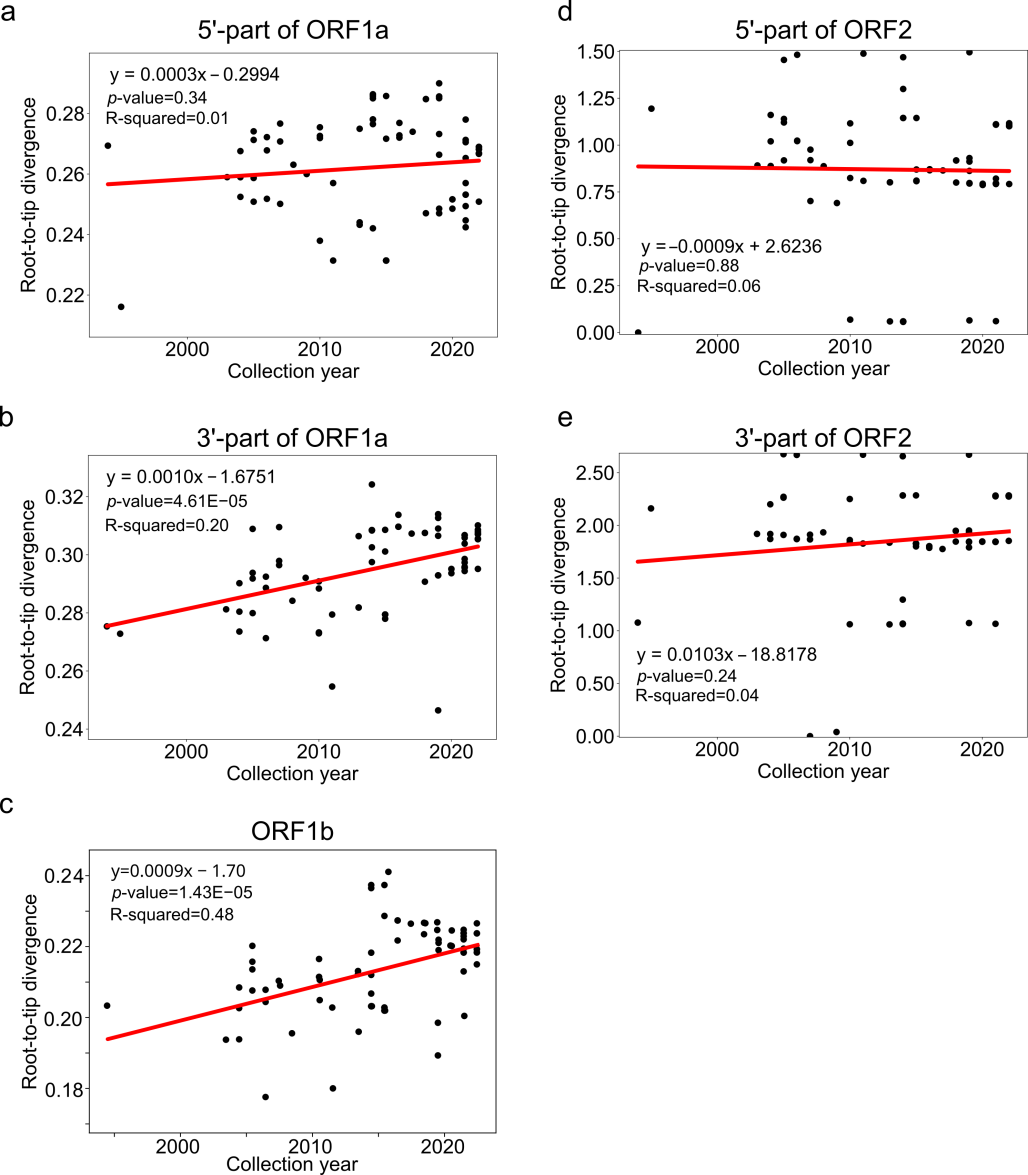
Evaluation of temporal signal across five recombination-free regions of human astroviruses. Root-to-tip regression analysis was performed in TempEst v.1.5.3 on ML phylogenetic trees for: 5′ half of ORF1a (**a**), 3′ half of ORF1a (**b**), ORF1b (**c**), 5′ end of ORF2 (**d**), and 3′ end of ORF2 (**e**). Each panel shows the linear regression fit (red line), regression equation, *p*-value for regression slope. The regression slope indicates substitution rate (substitutions/site/year).

To further identify the genomic regions suitable for molecular dating, we first assessed temporal signal in all recombination-free regions by regressing root-to-tip distances from maximum likelihood (ML) phylogenetic trees against collection dates (Figure 2). Only two regions in our dataset of complete human astrovirus genomes exhibited clock-like evolution: the 3′ end of ORF1a (regression slope *p* = 4.61 × 10^−5^, R^2^ = 0.20) and ORF1b (regression slope *p* = 1.43 × 10^−5^, R^2^ = 0.48). Bayesian estimation of temporal signal subsequently supported a heterochronous model (which incorporates actual sampling dates), confirming the presence of temporal signal in these datasets (Table 3).

Substitution rates inferred by BEAST v2.7.7 were 2.35 × 10^−3^ s/s/y for 3′-part of ORF1a and 2.14 × 10^−3^ s/s/y for ORF1b, values that are consistent with those typical of positive-sense RNA viruses. The time to the most recent common ancestor (tMRCA) was estimated at 251 years before present (ybp) for 3′-part of ORF1a and 208 ybp for ORF1b, with overlapping 95% highest posterior density (HPD) intervals for both rates and tree heights (Table 4).

### 3.3. Estimating the Persistence of Recombinant Lineages

**Figure 3:**
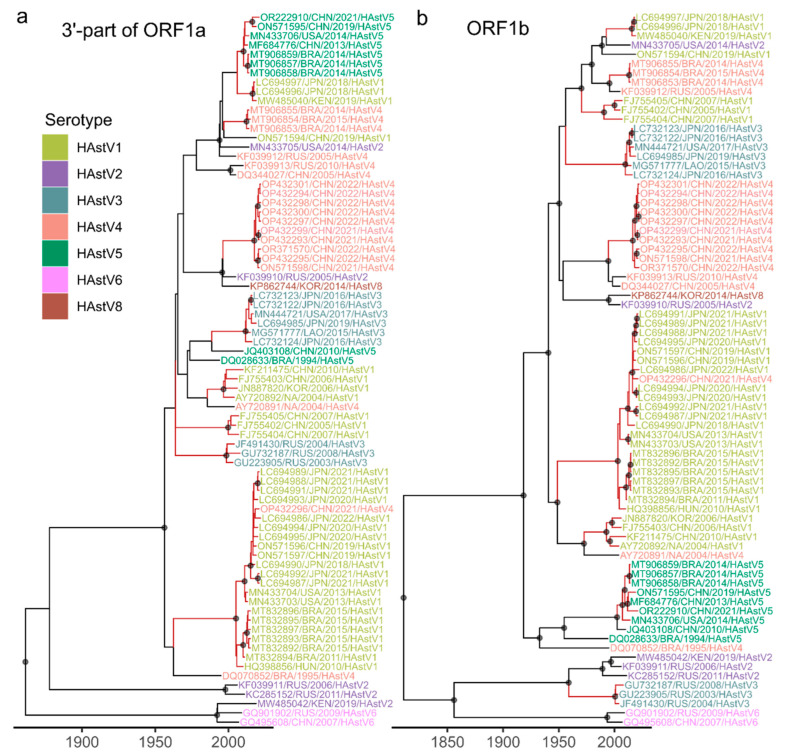
Inference of half-life of recombinant forms that occurred due to recombination between 3′-part of ORF1a and ORF1b genome regions of classical human astroviruses. Bayesian time-scaled trees were built using 3′-part of ORF1a (**a**) and ORF1b (**b**) genome regions. In both trees, black circles indicate nodes with high support (posterior probability > 0.9). Branches of clades that coincide between the trees are colored red. Taxa labels are colored according to the serotype.

The inferred time-scaled phylogenies for 3′-part of ORF1a and ORF1b enabled dating the recombinant forms. Following the phylogenetic approach established in our previous work on foot-and-mouth disease virus [56], we define recombinant forms as virus lineages that originate from a recombination event and subsequently persist without undergoing additional detectable recombination. Such lineages can be identified through comparison of tree topologies inferred from different genomic regions, appearing as coinciding clades—well-supported groups of sequences that share identical topology across trees. To systematically identify these clades, we employed the RF-HL program, which adopts a bipartition-based approach for tree comparison. This method treats each tree as a set of bipartitions (branches) that split the leaf set into two groups, then searches for coinciding bipartitions that meet predefined support thresholds. Nested bipartitions corresponding to internal branches of the resulting coinciding clades are omitted to avoid redundancy. The lifetime of a recombinant form is calculated as the time between the time of its most recent common ancestor (tMRCA) and the collection date of its most recent isolate; the recombinant form half-life is then defined as the median lifetime across all identified recombinant forms. We searched specifically for bipartitions that were reliably supported, requiring at least 95% ultrafast bootstrap support in ML trees and posterior probability > 0.9 in Bayesian time-scaled phylogenies (Figure 3 and Figures S1–S3).

To date the recombinant form lifetimes, we compared two time-scaled phylogenies—for 3′-part of ORF1a and for ORF1b—with ML phylogenies inferred from other genome regions exhibiting minimal or no recombination: 5′-part of ORF1a and two parts of ORF2. The phylogenetic tree for the 5′-part of ORF2 showed limited resolution, with only 23 out of 75 branches achieving ultrafast bootstrap support above 95%; this region was therefore excluded from further analysis. As time-scaled phylogenies were available for both 3′-part of ORF1a and ORF1b, we dated each set of coinciding clades twice—first using 3′-part of ORF1a tree as the temporal reference, then using the ORF1b tree—allowing to assess the consistency of lifetime estimates across the reference trees.

**Figure 4:**
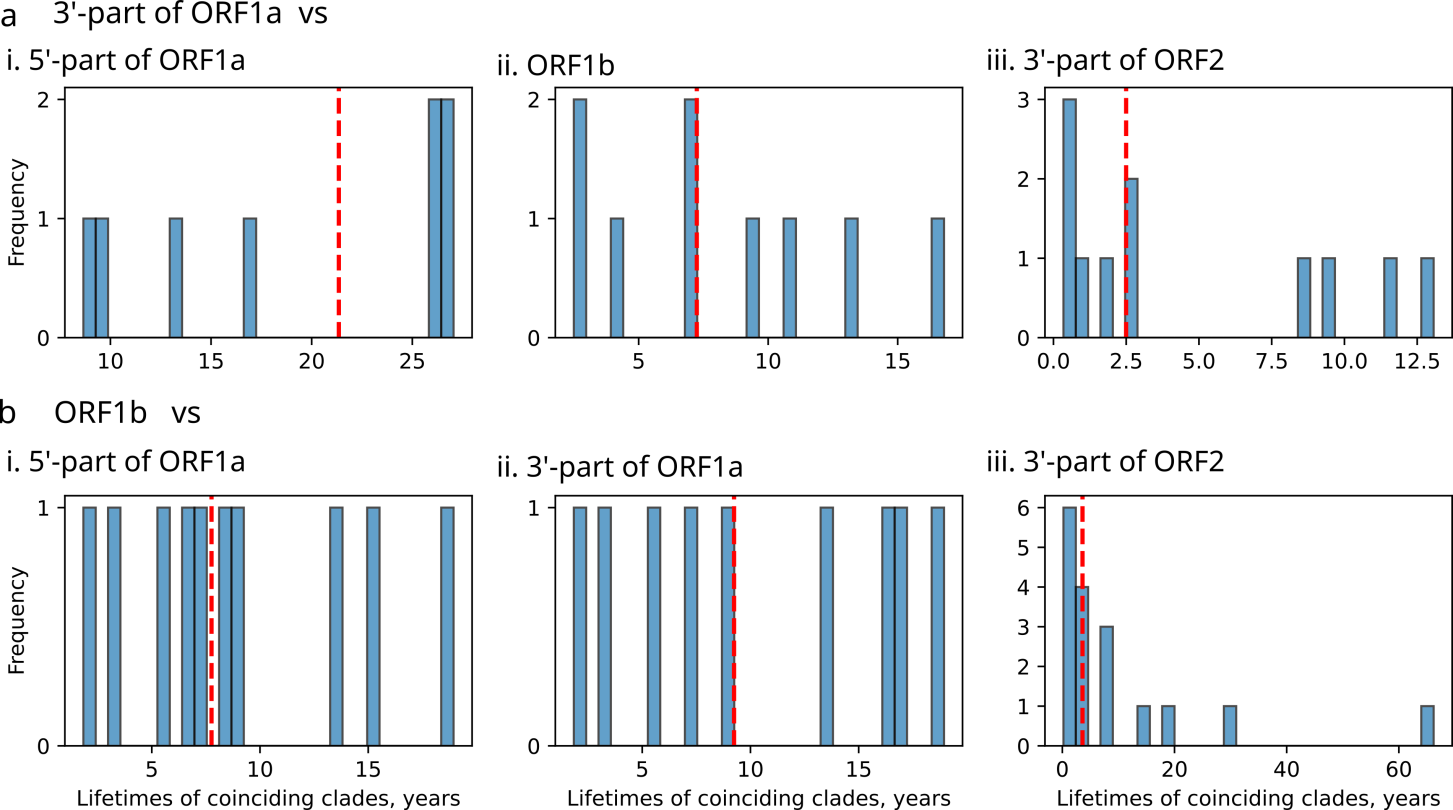
Distribution of lifetimes (time between tMRCA and the most recent isolate) of coinciding clades (recombinant forms) inferred from phylogenetic comparisons between Bayesian time-scaled phylogenies of reference regions with temporal signal, the 3′-part of ORF1a (**a**) and ORF1b (**b**) and trees from other genome regions: (**i**) 5′-part of ORF1a (ML tree), (**ii**) ORF1b (Bayesian phylogeny), (**iii**) 3′-part of ORF2 (ML tree). Dashed red line indicates recombinant form half-life (median of clade lifetimes).

**Figure 5:**
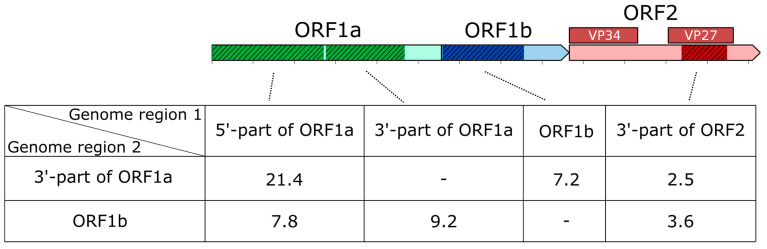
Half-lives of recombinant forms (in years) inferred from phylogenetic comparisons between genome regions involved in recombination. Top: Schematic representation of the human astrovirus genome, with regions used for phylogenetic comparison highlighted in brighter colors and marked with a diagonal pattern. Bottom: Median lifetimes of clades coinciding between Bayesian time-scaled phylogenies of the 3′-part of ORF1a and ORF1b (rows) and maximum likelihood trees of other genome regions (columns). For comparison of the 3′-part of ORF1a and ORF1b, the Bayesian phylogenies were utilized. Dashes indicate cells corresponding to the same tree in a row and a column.

Applying this approach, we found that lifetimes of coinciding clades ranged from 0.24 to 66 years, depending on the regions compared (Figure 4). The resulting half-lives of recombinant forms are summarized in Figure 5. Comparison between the 5′ and 3′-parts of ORF1a revealed the longest persistence of recombinant forms in circulation, with lifetimes ranging from 9 to 27 years (Figure 4a-i) and a half-life of 21.4 years (Figure 5). This extended persistence aligns with the rare recombination incidence observed between these regions (Figure 1). When comparing the 5′-part of ORF1a with ORF1b, the median lifetime of coinciding clades was nearly threefold shorter (7.8 years). Recombinant forms observed when comparing 3′-part of ORF1a and ORF1b exhibited half-lives of 7.2 years when dated using 3′-part of ORF1a as the reference and 9.2 years when using ORF1b, reflecting modest discrepancies in clade dating between the two reference trees (Figure 5).

**Figure 6:**
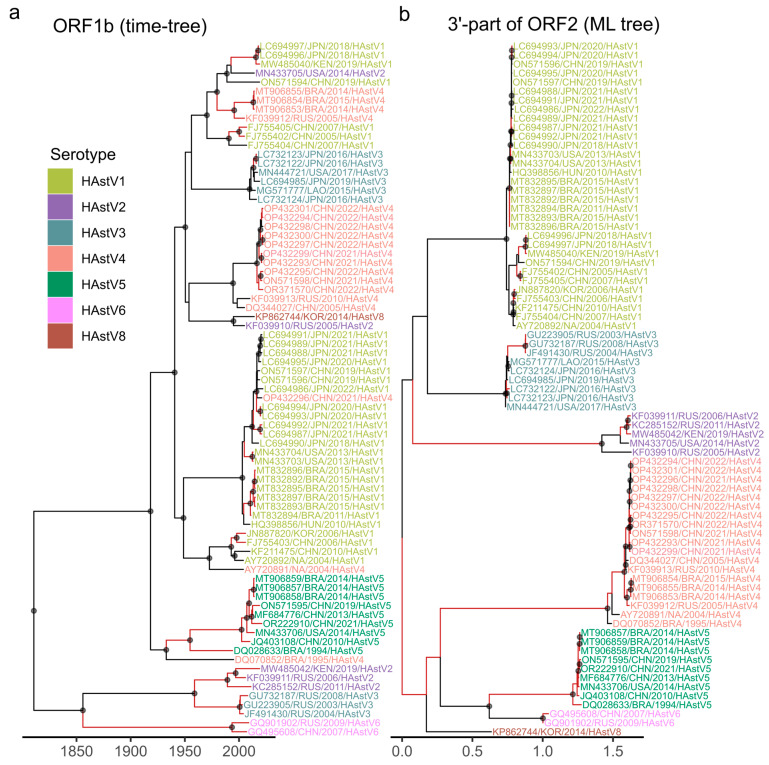
Inference of half-life time of recombinant forms that were made up by recombination between ORF1b and 3′-part of ORF2 genome regions of classical human astroviruses. (**a**) Bayesian time-scaled tree for ORF1b genome region. (**b**) ML phylogeny inferred using the 3′-part of ORF2 region. Black circles indicate nodes with high support (posterior probability > 0.9 in (**a**), ultrafast bootstrap >95% in (**b**)). Clades (bipartitions) that coincide between trees are colored red. Taxa labels are colored according to the serotype.

The highest degree of phylogenetic incongruence was observed between nonstructural regions (3′-part of ORF1a and ORF1b) and the structural ORF2 (Figure 6 and Figure S3). Sequences from different HAstV serotypes were extensively intermixed in trees built using nonstructural regions, indicative of frequent recombination between viruses from different serotypes (Figure 6). Subsequently, the highest number of recombinant forms could be identified between non-structural and structural genome regions (Figure 4). Comparisons of these genome regions also yielded the shortest half-lives of recombinant forms: 2.5 years for 3′-ORF1a versus 3′-part of ORF2 and 3.6 years for ORF1b versus 3′-part of ORF2 (Figure 5). These values are consistent with the recombination hotspot at the ORF1b/ORF2 junction and underscore the rapid turnover of lineages generated by recombination in this genomic region.

## 4. Discussion

Recombination is a well-established driver of genetic diversity in astroviruses, yet its patterns remain less explored than in other positive-sense RNA virus families. While multiple natural recombinants of classical human astroviruses (*Mamastrovirus hominis*) have been reported in field studies (Table 1), expanding availability of complete genome sequences has enabled a systematic evaluation of recombination patterns. Our analysis confirmed the single significant recombination hotspot located at the ORF1b/ORF2 junction, which was also observed at the genus *Mamastrovirus* level [23]. This finding aligns with a general pattern among positive-sense RNA viruses: frequent recombination between genomic regions encoding structural and non-structural proteins [55,57,58,59,60,61,62,63].

In addition to this canonical hotspot, several additional clusters of breakpoints were observed (i) within ORF1a, specifically in the 3′-end of region encoding the serine protease p27; (ii) near the ORF1a/ORF1b junction, within the p20 protein; and (iii) within ORF2 itself, with breakpoints clustering in the middle of the ORF2 (between the VP34 and VP25/VP27 encoding regions) and at the 3′ end corresponding to the acidic C-terminal domain. These observations are consistent with previously reported events (Table 1) and mirror patterns observed across the *Mamastrovirus* genus, where recombination was infrequent in ORF1b and in the ORF2 regions encoding VP34 [23].

Based on the recombination patterns identified in classical human astroviruses, five distinct genomic regions that were spared by recombination could be identified: the 5′-part of ORF1a, the 3′-part of ORF1a, the majority of ORF1b, and two segments of ORF2 corresponding approximately to the VP34 and VP25/VP27 coding regions (termed here the 5′-part of ORF2 and the 3-part of ORF2). The generation of recombinant variants in human astroviruses thus appears to occur through frequent recombination at the major ORF1b/ORF2 region hotspot, and lower-frequency recombination events at other genome locations, particularly at domain boundaries within ORF1ab and ORF2.

Consistent with previous reports (Table 1), we found that recombination breakpoints in ORF2 occur at domain boundaries, preserving the integrity of the functional cores of individual capsid proteins. This pattern resembles the modular evolution observed in the coronavirus spike gene, where recombination occurs between protein domains but not within them, enabling the exchange of functional modules while maintaining the overall protein architecture [59,64].

To investigate the temporal dynamics of these recombination events, we first established a reliable timescale for astrovirus evolution. This required identifying genomic regions that were spared by recombination and retained a sufficient temporal signal for molecular clock analysis. Only two genome regions—3′-part of ORF1a (~1500 nt in length) and a fragment of ORF1b (~1100 nt in length)—exhibited robust temporal signal and yielded comparable substitution rates of 2.35 × 10^−3^ s/s/y and 2.14 × 10^−3^ s/s/y, respectively. These values are lower than 3.7 × 10^−3^ s/s/y for 600 nt fragment of ORF1a and 4.1 × 10^−3^ s/s/y for 430 nt fragment of ORF2 reported by Babkin et al. [8]. However, the analysis by Babkin et al. was based only on 16 complete genomes and did not include serotype 2 and most serotype 4 sequences, many of which have since become available and were incorporated into our study. More recently, Zhou et al. estimated a substitution rate of 4.51 × 10^−4^ s/s/y for the ORF2 [7]. In our dataset, which comprised complete genomes of classical human astroviruses, the complete ORF2 was not usable for phylogenetic analysis due to multiple recombination events within, and recombination-free fragments of ORF2 did not exhibit a sufficient temporal signal to permit reliable rate estimation. Therefore, it is not clear if there is a true difference in substitution rates between ORF1 and ORF2.

To determine the lifetimes of recombinant forms in human astroviruses, we employed a phylogenetic approach. A key advantage of this method is that it does not rely on pre-defined genotype or lineage classifications. While such classifications are well established for some virus groups—such as the dual nomenclature for polymerase and capsid proteins in noroviruses [65]—no comparable nomenclature exists for human astroviruses. Moreover, given the complex evolutionary dynamics of astroviruses, any attempt to establish a similar classification would likely be arbitrary.

The phylogenetic approach bypasses some limitations but is a subject to others. It requires reliable phylogenies with robust branch support. In practice, inferred trees often lack high bootstrap support across all branches, especially when analyzing datasets with closely related sequences. For clades with highly similar sequences, low bootstrap support precludes precise topology comparisons and thus detection of recombination. To address this, we employed a softer definition of a recombinant form that does not require exact topological matches within clades but instead identifies highly supported branches that are congruent between trees. However, even with this relaxed definition, comparisons become uninformative when most branches in the tree lack high bootstrap support.

The problem of low bootstrap support was aggravated by the need to partition the genome. Dividing the genome into regions with no internal recombination breakpoints is necessary because recombination can disrupt tree topology. However, this partitioning may yield short genomic fragments with limited phylogenetic resolution. Among the five genome blocks involved in recombination that showed no internal recombination, the phylogenetic tree for the 5′-part of ORF2 exhibited especially low branch support (only 30.6% of branches with >95% bootstrap), making phylogenetic comparisons uninformative.

The estimation of recombinant form lifetimes may vary depending on the sequence dataset and the reference time-scaled phylogeny used. The median lifetimes of clades that were congruent between the 3′-part of ORF1a and ORF1b phylogenies differed depending on which tree served as the dating reference. Specifically, half-life estimates for recombinant forms varied by approximately two years–7.2 years when calibrated against the ORF1a phylogeny versus 9.2 years when using the ORF1b phylogeny. Recently, we demonstrated for FMDV that, across different sequence subsamples, the standard deviation of recombinant form half-lives ranged from 0.79 to 1.58 years [56]. Thus, the observed two-year difference between estimates falls within the range of expected variability given the inherent uncertainties of molecular clock dating.

Even though molecular dating of recombination events was rather consistent between the 3′-part of ORF1a and ORF1b, a significant limitation comes from the sampling bias. Serotype coverage was very uneven among the published sequences (Table 2). Moreover, most genomes were sampled over the last five years, and mainly in just a few countries. One can speculate how a better sampling can either increase (by being able to trace circulation of lineages for a longer time) or decrease (by detecting more recombination events) the observed recombinant form half-lives. However, it is unlikely that better sampling would yield radically different recombination rate estimates. In any case, the timing reported here should be taken with caution and interpreted more as an order of magnitude than as precise values.

The patterns and dynamics of recombination in classical human astroviruses correspond very well to those in other enteric RNA viruses. For enterovirus echovirus 30, turnover of recombinant forms made up of structural and non-structural genome regions occurs every 3–5 years, for echovirus 9 approximately every year, and for echovirus 11 every 10 years [66,67]. In enterovirus A71, the half-life of recombinant forms varies by genotype (ranging from 6 to 10 years), with some prevalent recombinant forms persisting without detectable recombination for several decades [68]. Among noroviruses of the GI and GII genogroups—the major causes of adult diarrhea—recombinant forms’ half-lives were longer, estimated at 8 and 10 years respectively, yet remain within the same order of magnitude as those of enteroviruses [55].

In human astroviruses, the half-lives of recombinant forms involving structural and nonstructural regions fell within this same range: 2.5 years for recombinants between the 3′-part of ORF1a and the 3′-part of ORF2, and 3.6 years for those between ORF1b and the 3′-part of ORF2. However, the more complex recombination landscape of astroviruses also includes exchanges confined to the nonstructural region, which exhibited markedly different temporal dynamics. In contrast to the short-lived recombinant forms arising from exchanges between nonstructural and structural regions (2.5–3.6 years), those defined by recombination events within the nonstructural part of the genome exhibited markedly longer persistence. Recombinants involving exchanges between the 5′- and 3′-parts of ORF1a had a half-life of 21.4 years, while those between ORF1a and ORF1b showed intermediate half-lives of 7.2–9.2 years. Therefore, recombination in enteric RNA viruses follows not just similar patterns [69], but also comparable dynamics on a global scale.

## Figures and Tables

**Table 2:** The number of sequences of each serotype in the refined dataset.

HAstV Serotype	Number of Complete Genomes
Serotype 1	32
Serotype 2	5
Serotype 3	9
Serotype 4	19
Serotype 5	9
Serotype 6	2
Serotype 7	0
Serotype 8	1

**Table 3:** Results of BETS analysis for two genome regions with a detectable temporal signal. BETS analysis compares the fit of a heterochronous model (utilizing true sampling dates) to an isochronous null model (all dates set equal). Marginal likelihood estimates were calculated using the nested sampling algorithm implemented in BEAST2 v2.7.7. A log Bayes factor greater than 3 indicates strong support for the presence of temporal signal.

	3′-Part of ORF1a	ORF1b
Isochronous model	−11,916.34	−8013.75
Heterochronous	−11,779.94	−7969.57
Log Bayes Factor	136.40	44.10

**Table 4:** The evolutionary parameters of human astroviruses estimated for recombination free regions using BEAST2 v2.7.7.

Genome Region	Substitution Rate [95% HPD Confidence Interval] × 10^−3^, s/s/y	tMRCA [95% HPD Confidence Interval], Years
3′-part of ORF1a	2.35 [1.85–2.86]	251.38 [195.83–313.11]
ORF1b	2.13 [1.52–2.78]	208.04 [145.07–278.21]

## Data Availability

The data presented in this study (sequence names and metadata, alignments, phylogenetic trees) are openly available at https://github.com/v-julia/HAstV_recombination_dynamics (accessed on 15 March 2026).

## Supplementary Materials

The following supporting information can be downloaded at: https://www.mdpi.com/article/10.3390/microorganisms14040857/s1, Table S1: Human astrovirus GenBank entries used in this study and corresponding metadata. Table S2: Model selection for BEAST analysis. Figure S1: Inference of half-life time of recombinant forms that were made up by recombination between 3′-part of ORF1a and 5′-part of ORF1a genome regions of classical human astroviruses. (A) Bayesian time-scaled tree for 3′-part of ORF1a genome region. (B) ML phylogeny inferred using the 5′-part of ORF1a region. Black circles indicate nodes with high support (posterior probability > 0.9 in (A), ultrafast bootstrap > 95% in (B)). Clades (bipartitions) that coincide between trees are colored red. Taxa labels are colored according to the serotype. Figure S2: Inference of half-life time of recombinant forms that were made up by recombination between ORF1b and 5′-part of ORF1a genome regions of classical human astroviruses. (A) Bayesian time-scaled tree for ORF1b genome region. (B) ML phylogeny inferred using the 5′-part of ORF1a region. Black circles indicate nodes with high support (posterior probability > 0.9 in (A), ultrafast bootstrap > 95% in (B)). Clades (bipartitions) that coincide between trees are colored red. Taxa labels are colored according to the serotype. Figure S3: Inference of half-life time of recombinant forms that were made up by recombination between 3′-part of ORF1a and 3′-part of ORF2 genome regions of classical human astroviruses. (A) Bayesian time-scaled tree for 3′-part of ORF1a genome region. (B) ML phylogeny inferred using the 3′-part of ORF2 region. Black circles indicate nodes with high support (posterior probability > 0.9 in (A), ultrafast bootstrap > 95% in (B)). Clades (bipartitions) that coincide between trees are colored red. Taxa labels are colored according to the serotype.

## Abbreviations

The following abbreviations are used in this manuscript:

CDS	Coding sequence
HAstV	Human astrovirus
HPD	Highest posterior density
ML	Maximum likelihood
ORF	Open reading frame
tMRCA	Time to the most recent common ancestor
