# Supplementary figures and images for: Complex Recombination Landscape and Lineage Turnover in Classical Human Astroviruses
Source: Microorganisms. 2026 Apr 10;14(4):857. doi: 10.3390/microorganisms14040857 (PMC13118342; doi:10.3390/microorganisms14040857)

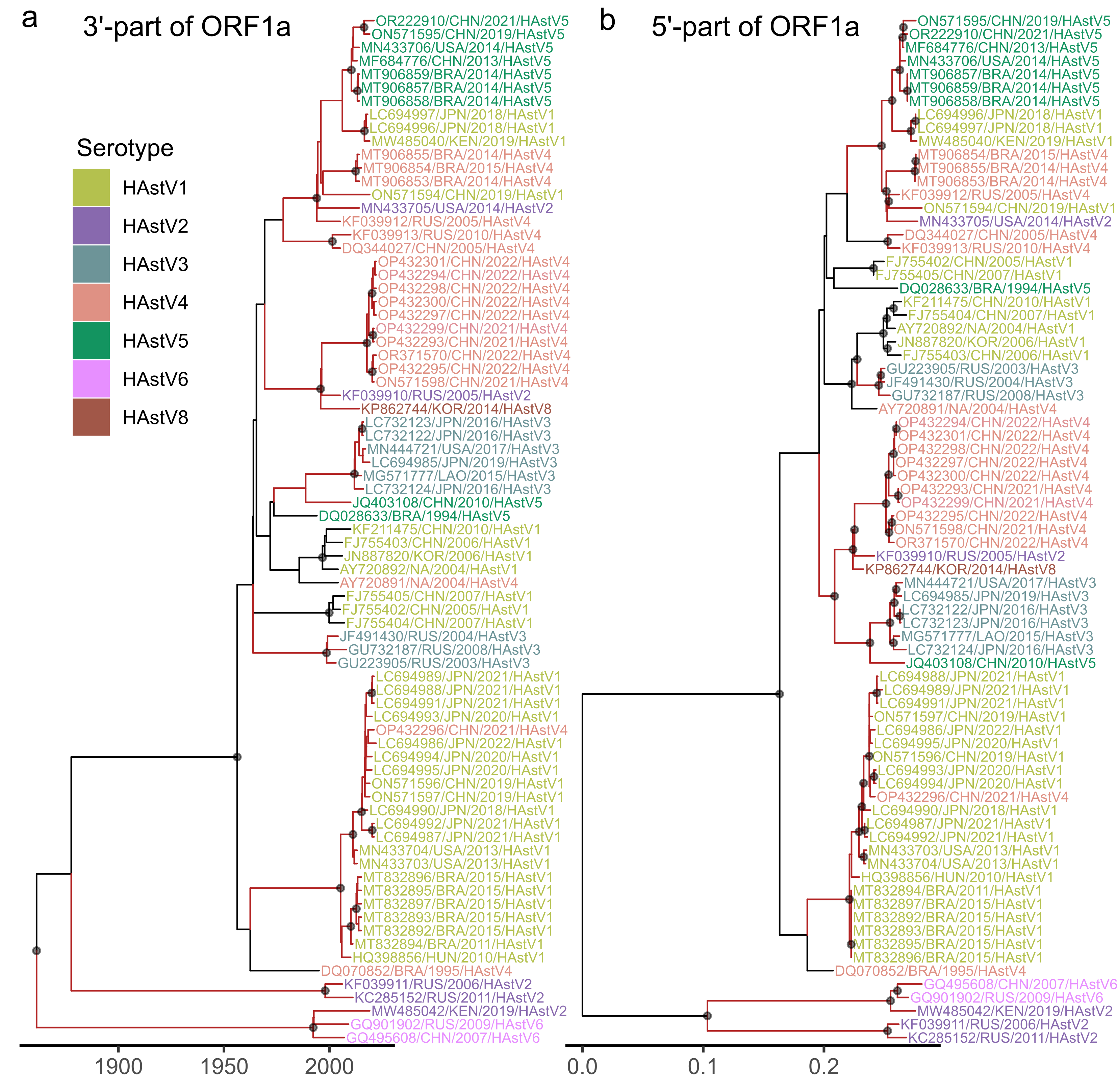

Supplement: Supplementary file 1 [file microorganisms-14-00857-s001.zip › FigureS1.png]

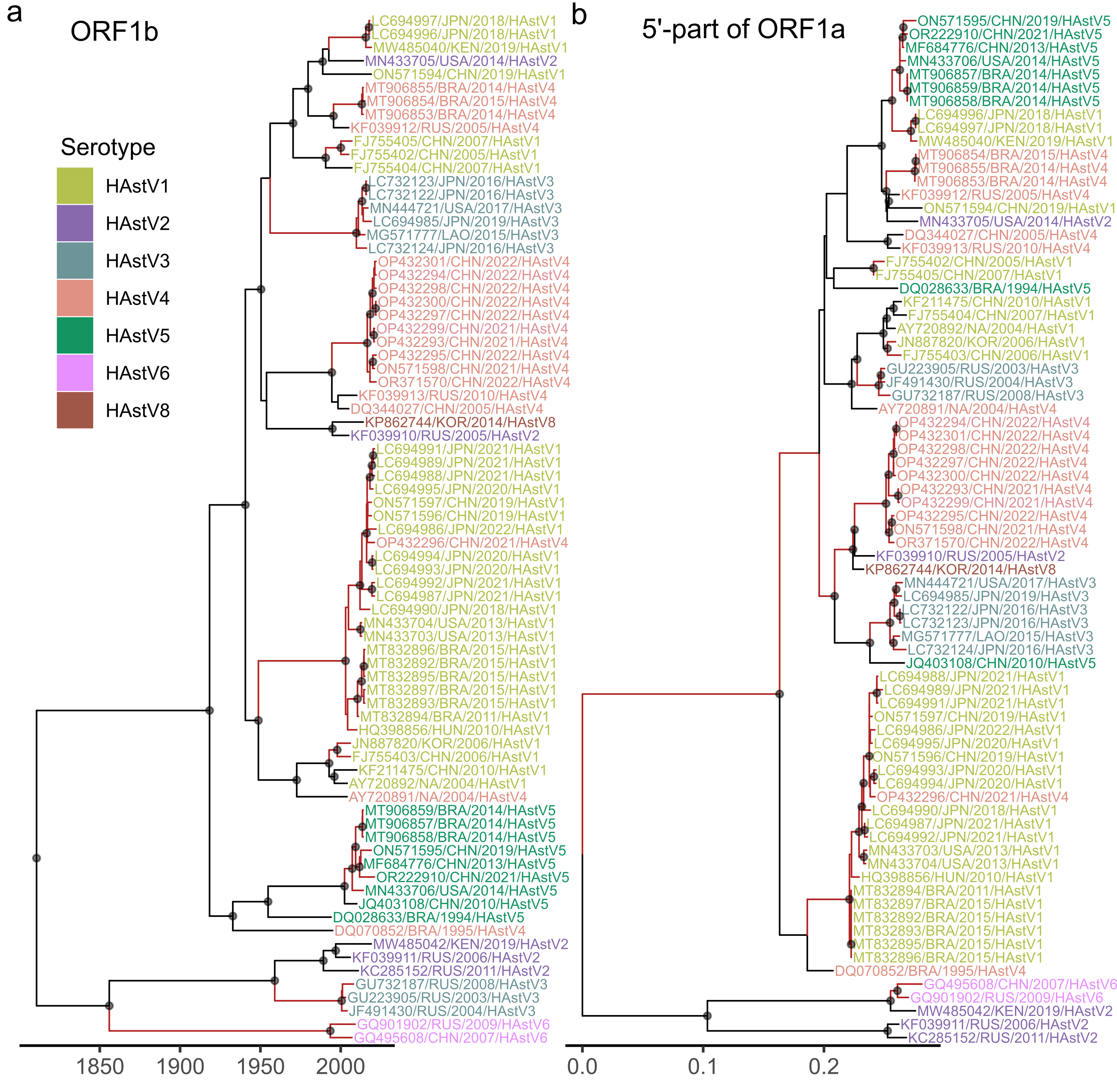

Supplement: Supplementary file 1 [file microorganisms-14-00857-s001.zip › FigureS2.png]

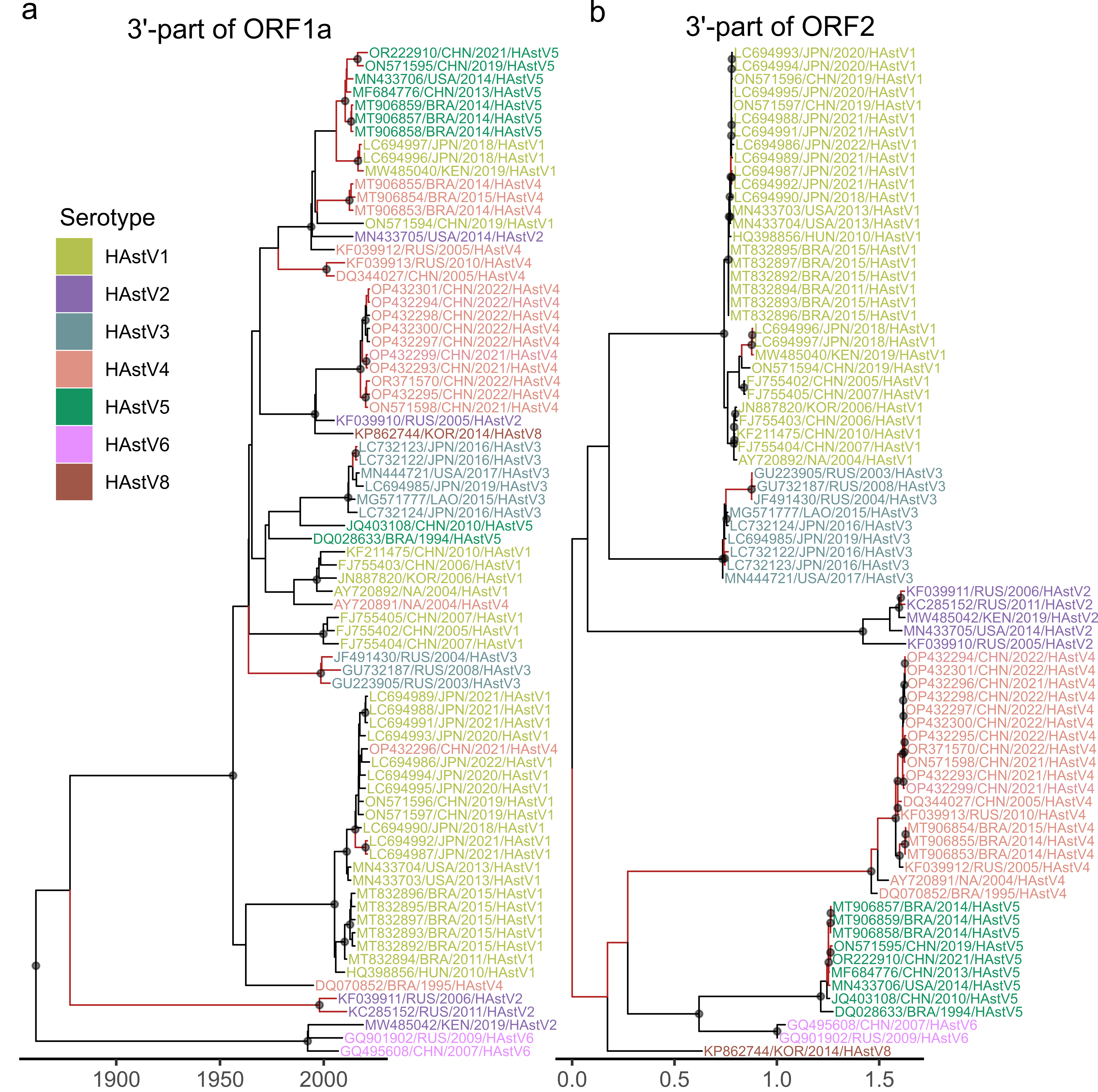

Supplement: Supplementary file 1 [file microorganisms-14-00857-s001.zip › FigureS3.png]
